# Organized Sport Activities of 11 to 15-Year-Old Adolescents: Trends from 2010–2018 and Socioeconomic Context

**DOI:** 10.3390/ijerph19106074

**Published:** 2022-05-17

**Authors:** Vladimir Hobza, Marek Maracek, Zdenek Hamrik

**Affiliations:** Department of Recreation and Leisure Studies, Faculty of Physical Culture, Palacky University Olomouc, 779 00 Olomouc, Czech Republic; marek.maracek@upol.cz (M.M.); zdenek.hamrik@upol.cz (Z.H.)

**Keywords:** leisure time, organized sport activities, socioeconomic status, adolescents, HBSC

## Abstract

The aim of this paper was to analyse the trends in the participation of Czech adolescents in organized sport activities in relation to the socioeconomic status (SES) of their families. The sample consisted of 11, 13 and 15-year-old children (N = 4425 (2010), 10,361 (2014) and 13,377 (2018)); the data were collected as part of the HBSC Study in the Czech Republic. The findings suggest that adolescents from affluent families tend to participate more often in organized sports—both team activities and individual activities. Fifteen-year-old adolescents from families with a high SES are 3.01 times more likely to participate in individual organized sports than adolescents from low-SES families. The gap between participation in sport activities increases with the children’s age but does not change significantly during the observed period. The findings suggest that public health policy should be oriented towards children from less-affluent families.

## 1. Introduction

Leisure-time activities play an important role in the context of adolescents’ health and wellbeing [1,2] and are linked to numerous positive developmental outcomes such as improved learning results [3,4] or lower inclination towards high-risk behavior [5]. Badura [6] states that almost three-quarters of children (73.5%) in the Czech Republic are involved in organized leisure activities in the fifth grade, compared to more than half (57%) in the ninth grade. Thus, participation in leisure-time activities seems to be one of the highest across countries [7].

From the perspective of health and lifestyle, participation in organized leisure activities is perceived very positively, as it develops adolescents’ human, social and cultural capital [8]. Participation in organized leisure activities is considered to be one of the most important factors that influence adolescents’ healthy development [5]. These activities provide opportunities for adolescents to improve their social, physical and intellectual skills [9], support the development of self-control, adaptation and problem-solving skills [4] and, in group interactions, bring social recognition and reward. In this way, they can effectively prevent risk behaviour [5]. Adolescents who spend their time in organized activities show greater life satisfaction, better health [6], academic achievement and attitude towards school [10] and lower degrees of substance use [11,12] and asocial or delinquent behaviour [13] compared with those who are not involved in organized activities.

However, participation in organized activities is not the only crucial factor in the context of leisure and impact on children’s health [14]. An important role is played by the so-called dimensions of (organized) leisure activities including the type of the activity and the breadth, frequency and duration of involvement [5]. All of these dimensions may affect the outcomes of subjectively perceived health brought about by participation in organized leisure activities [5,15].

One of the most common types of leisure-time activities in which children and adolescents participate are sport activities (SAs) [16]. SAs therefore play a key role in leisure activities, not to mention their positive psychological and social impact in children and adults [17] as well as numerous positive health outcomes [3,6]. Adolescents who spend their time in an active way show lower body weight fluctuations and have a lower risk of the later onset of non-communicable diseases [18]. SAs also stimulate better performance in mental tasks and decrease anxiety, stress and depression [19]. An important contribution to an overall feeling of health is the interaction with peers and establishing friendships. SAs provide an ideal space for different forms of social interaction and friendship. Friendship between adolescents develops social competences as well as a sense of safety, support and happiness [20].

At the same time, maintaining a sufficient number of physical activities is key in the development of the individual, which can have a significant relationship to health and lifestyle [21]. Moreover, it is also important to point out that the effects of SAs vary by the dimensions of the SAs, such as breath of involvement (one sport activity/more sport activities) and the intensity and duration of engagement in such sport activity [5]. In addition, it seems that different kinds of SAs have different effects. For example, Badura et al. [6] discovered that individual and team sport activities showed different associations with life satisfaction, self-rated health or feeling low in adolescents.

To conclude, the majority of the research suggests that SAs play a key role in adolescents’ heath, wellbeing and healthy development. Moreover, there is a large volume of published studies describing the beneficial role of SA in children and adolescents [5,10,11,12]

One of the key influences in adolescents’ participation in leisure-time activities is socioeconomic status (SES). A low SES generally reduces participation in SAs in both boys and girls across all ages [22,23]. Socioeconomic status can be described as “a broad concept that refers to the placement of persons, families, households and census tracts or other aggregates with respect to the capacity to create or consume goods that are valued in our society” [17]. The socioeconomic status of adolescents depends on the status of their parents and can be measured by non-intrusive and easy-to-answer questions [24]. Generally, socioeconomic inequalities significantly influence child and adolescent health; thus, it is crucial to provide sufficient evidence-based research to effectively propose health-promoting public policies. The Czech Republic belongs to the set of countries with the lowest socio-economic inequalities [25] and is considered a developed country by the UN classifications [26]. Its level of income inequality, as measured by the Gini Index by the World Bank, ranks the Czech Republic as the country with the second-lowest inequality based on 2018 data [25].

Contrary to this, the issue of inequalities in sport participation is a widely discussed topic. A study presented by Sigmund et al. [1] suggests that family socioeconomic status may play a significant role in the physical activity, screen time behaviours, obesity and sport participation of Czech adolescents. However, to our best knowledge, a more detailed analysis of socio-economic inequalities in access to sporting activities for children and adolescents is lacking. Thus, this paper aims to address the following research question: What is the correlation between socioeconomic status and participation rate in team and individual sport activities in 11, 13 and 15-years old Czech adolescents from 2010 to 2018? The data were collected as part of the Health Behaviour in School-Aged Children (HBSC) study in the Czech Republic.

## 2. Materials and Methods

### 2.1. Sample Size and Data Collection

The nationally representative sample of Czech girls and boys from the Czech HBSC study was used. The sampling was performed in accordance with HBSC study protocol [27], which was developed and validated by researchers of the participating countries and allows for international comparison. Age was calculated by given birth date and only children of age categories 11, 13 and 15 years were included in the dataset. These categories, respectively, generally correspond with the 5th, 7th and 9th grade of Czech elementary schools and their equivalents. The data were collected in 2010, 2014 and 2018. The first two data collections were paper-based. The 2018 data collection was electronic, using the Unipark^®^ online tool by Tivian XI GmbH, Köln, Germany.

The data were obtained from the nationally representative HBSC sample which includes randomly selected schools, after stratification for regions. The 2010 data collection consisted of 4425 participants (2280 girls, 2145 boys) from 86 schools, the 2014 collection had 10,361 participants (5268 girls and 5093 boys) from 243 schools and in the last 2018 collection, data from 13,377 children (6569 girls, 6808 boys) from 234 schools were collected. The dataset was checked for errors and cleared in accordance with the HBSC study protocol [27]. This paper includes only data that included both all items relevant for the calculation of the Family Affluence Scale (FAS) and all answers with regards to SA. This led to a reduction of the final sample to 4148 participants for 2010, 10,263 for 2014 and 12,862 for 2018. The data collection was conducted with the approval of the Ethics Committee of the Faculty of Physical Culture, Palacky University Olomouc (ethical code: 16/2014).

### 2.2. Measures

We used the Family Affluence Scale (FAS) for the identification of socioeconomic differences. The tool was developed and validated by the HBSC network [28,29,30]. It uses six simple-to-answer questions, which were selected from 21 pilot questions, as tools with adequate validity and reliability to identify the family socioeconomic status. The resulting sample was then stratified into three subgroups of Low (0–20 percentile), Medium (21–97 percentile) and High (80–100 percentile) socioeconomic status. The FAS showed good correlation with the net household income given by parents (r = 0.53–0.55) and high test-retest reliability (r = 0.90) [31]. The scale was also validated using macroeconomic measures and proved to be sufficiently reliable [28,29,30] in the Czech Republic [32].

In 2018 and 2014, participation in SAs was assessed using the following validated question: *In your leisure time, do you do any of the following organized activities? Organized activities should be understood as activities performed in a sports club or a different club or organization.*

*(1)*  *Organized team sports (for example football, volleyball, floorball) Answers: Yes/No*
*(2)*  *Organized individual sports (for example tennis, gymnastics, karate) Answers: Yes/No*

In 2010, participation in organized sports was measured by a different set of questions which did not include a Yes/No answer but frequency instead. *(1) Twice a week or more (2) Once a week (3) Several times a month (4) Never*. The Yes/No answer was extracted by using cut-off values. The Yes/No cut-off was placed between answers 1 and 2, which, based on our judgement, should represent regular participation in sport.

### 2.3. Statistical Analyses

The analysis was conducted using IBM SPSS^®^ 21 (IBM Corp., Armonk, NY, USA) and the figures and tables were further processed by MS Excel^®^ 2019 and MS Word^®^ 2019. First, we described the sample and participants’ involvement in organized team and individual sport activities by FAS categories and age groups. Second, we assessed the dependence of organized sport activities on FAS using a non-parametric Spearman regression to verify the statistical significance of family affluence on participation in organised sport activities. Next, we identified the odds ratios (OR) by running a binary logistic regression on the sample. Finally, we adjusted the logistic regression model for age and gender correlates. To identify more details, we decided to analyse the time series for the identification of possible trends. The statistical significance of trends can be assessed by observing overlapping confidence intervals of odds ratios.

## 3. Results

### 3.1. Involvement in Organized Sport Activities

The participation rate in team and individual SAs is presented in Table 1.

### 3.2. Correlations

The Spearman correlation shown in Table 2 identified low but significant levels of correlation. The correlation was slightly higher in individual SA in 2018 and in team SA in 2014 with no clear indication of a trend.

### 3.3. Odds Ratios

The odds ratios in Table 3 show a very significant (*p* < 0.01 or less) difference in SA participation in individual sports. Furthermore, the odds ratios increase in each year in all of the age categories (11, 13, 15) but the confidence intervals of both ending periods overlap, suggesting that the trend cannot be identified as statistically significant.

In team sports, there is only one age category where we cannot identify a significant difference between the odd ratios in high- and low-FAS 11-year-old children in the 2010 data. However, in 2014 and 2018, all of the odd ratios in the sample were already statistically significant.

## 4. Discussion

The results show that higher family affluence was significantly correlated with participation in both team and individual organized activities in 11, 13 and 15-year-old adolescents in the Czech Republic based on the HBSC 2010–2018 data. The findings remained significant after adjustments for age and gender. These results are consistent with those of other studies [1,7,33], showing a positive relation between socioeconomic status and sport participation in Czech adolescents. This is consistent with similar foreign studies [17,22,34]. Although the overall trend is one of rising participation in organized sport activities, we can identify an increasing diversity between sport participation in adolescents from low- and high-affluence families.

Higher OR differences between low and high FAS in individual sports could be reasoned by the fact that individual SAs generally tend to be more expensive, due to the need for the personal approach by a coach or trainer, and therefore less affordable to low-income families. On the contrary, team sport activities, where the coach cost is distributed among all course participants, tend to be generally cheaper.

The results of this research support the idea that the difference between low- and high-FAS children’s participation in SAs increases during the observed period in some age categories (see Table 3). Nevertheless, there seems to be no statistically significant difference between the 2010, 2014 and 2018 data, as suggested by the overlap in confidence intervals in OR. In contrast, the overall participation in SAs decreases strongly with age, notably for 15-year-old adolescents. The present findings seem to be consistent with other research [1,6] which concluded that Czech adolescents tended to participate less in organised leisure-time activities as they moved from grade 5 to grade 9. This paper further identified in more detail that the tendency was especially notable in the low-FAS category of adolescents and individual sport activities. The odds ratio showed that high-FAS 15-year-old adolescents were more than three times as likely to participate in organized individual sport activities as their peers from less-affluent families (high vs. low FAS). This effect was not significant in 11-year-old participants for team activities, probably due to the fact that team activities tend to be generally cheaper and more accessible, in some cases regardless of FAS. Furthermore, this can be emphasized by the fact that younger children are generally less demanding than when they grow into adolescence.

### 4.1. Strengths and Limitations

The key strengths of this study are its long timespan and a large nationally representative sample collected using the well-established HBSC methodology. The study has further deepened and quantified our understanding of FAS concerning participation in team and individual SA. When discussing the findings of this paper, its limitations have to be considered as well. An obvious limitation of the research is the use of a cross-section design and self-reported participation in SAs without independent objective measurement. Another limitation of the study is the absence of a qualitative indicator and simplification of sport participation in dichotomous values. In 2010, participation in organized activities was measured by a different set of questions which did not include Yes/No answer but frequency instead. Therefore, we had to use a cut-off value to extract Yes/No answers. This paper used a cut-off value between 1 and 2. By placing the cut-off between answers 2 and 3, we gained much higher participation, especially with regards to team sport activities.

The results are limited to pre-COVID data, but the HBSC study continues and a new round of data collection is planned for 2022 which may shed light on the impact of the COVID-19 pandemic on the SAs of children and adolescents and the role of FAS and/or other factors. The pandemic not only limited participation in SAs in order to control the spread of the disease [35] but also brought an additional economic burden which had a relatively hard impact on low-income families. Therefore, we may expect a deepening of the differences in SA participation based on FAS, but further research and data are needed.

### 4.2. Implications

These findings bring implications for policy in the promotion of physical activity and sports. Even in countries where income inequalities are relatively low, the practical impact on sport participation is not negligible. Based on the findings, public policy should be more focused on low-income and low-FAS families as their participation in both team and individual sport activities is relatively low. Physical inactivity in adolescence is widely considered a public health issue [3,4,16,18]; therefore, health promotion policy should be primarily focused on supporting children from less-affluent families. This is in line with other studies in the field, which have also concluded that policies should support adolescents’ participation in organized leisure-time activities, with a specific focus on adolescents living in poverty [7]. Thus, policies by local authorities and sport clubs in low-income areas that provide economic assistance to low-income families with children who wish to participate in sports (such as reducing membership fees or costs for parents) should be encouraged [31]. In the Czech Republic, this might be supported by adequate policies and programs provided by the local governments and municipalities. Another option might be the better engagement of leisure centers (called Centres for Children and Youth in the Czech Republic) or schools to provide more sport opportunities for youth and adolescents.

## 5. Conclusions

In this study, the data show a statistically significant correlation between the team and individual SAs of Czech adolescents and family affluence in the given timeframe. The dependence does not significantly increase during the observed period, but increases with school grade. This could be an evidence-based impulse for policymakers, local governments and sport clubs to focus on the promotion of physical activity and sport for less-affluent families, especially in the area of individual sports.

## Figures and Tables

**Table 1:** Description of the study population: rates of respondents’ involvement in organized team and individual sport activities for data collected in 2010, 2014 and 2018, Health Behaviour in School-Aged Children study (HBSC) in the Czech Republic.

		Participation in Organized Activities (%)			
Activity	Age	FAS	2010	2014	2018
Team	11	Low	34.54%	44.68%	46.06%
		Medium	36.99%	52.13%	53.16%
		High	41.69%	58.32%	57.45%
	13	Low	25.74%	45.24%	41.67%
		Medium	33.13%	48.63%	48.51%
		High	40.74%	58.20%	56.04%
	15	Low	18.94%	35.86%	29.31%
		Medium	25.89%	42.54%	39.89%
		High	32.76%	48.74%	46.10%
Individual	11	Low	31.22%	24.57%	33.18%
		Medium	41.16%	33.16%	43.28%
		High	47.45%	46.35%	52.49%
	13	Low	30.58%	25.33%	26.26%
		Medium	34.73%	29.86%	35.39%
		High	42.33%	38.11%	47.45%
	15	Low	19.74%	19.04%	20.25%
		Medium	30.94%	25.54%	29.65%
		High	35.23%	37.55%	43.11%

**Table 2:** Spearman correlation between FAS category (1–3) and sport participation (yes/no).

		2018	2014	2010
Individual sports	Corr. coef.	0.121 **	0.084 **	0.087 **
Team sports	Corr. coef.	0.080 **	0.122 **	0.097 **

** *p* < 0.001.

**Table 3:** Odd ratios—sample divided into 3 age categories adjusted for age and gender.

	Age Category	FAS	2010	2014	2018
			Adjusted for Age, GenderOR (95% CI)	Adjusted for Age, GenderOR (95% CI)	Adjusted for Age, GenderOR (95% CI)
Team sports	11	low	1.00 (ref)	1.00 (ref)	1.00 (ref)
		medium	1.12 (0.80–1.57)	1.31 ** (1.1–1.56)	1.28 ** (1.07–1.52)
		high	1.42 (0.99–2.04)	1.71 *** (1.37–2.13)	1.49 *** (1.18–1.88)
	13	low	1.00 (ref)	1.00 (ref)	1.00 (ref)
		medium	1.37 (0.96–1.97)	1.14 (0.96–1.37)	1.31 ** (1.09–1.56)
		high	1.75 ** (1.18–2.59)	1.68 *** (1.36–2.08)	1.74 *** (1.4–2.16)
	15	low	1.00 (ref)	1.00 (ref)	1.00 (ref)
		medium	1.37 (0.94–2.00)	1.30 ** (1.08–1.56)	1.52 *** (1.26–1.84)
		high	1.81 ** (1.20–2.74)	1.62 *** (1.3–2.01)	2.01 *** (1.59–2.55)
Individual sports	11	low	1.00 (ref)	1.00 (ref)	1.00 (ref)
		medium	1.55 * (1.1–2.18)	1.56 *** (1.29–1.89)	1.62 *** (1.35–1.95)
		high	1.96 *** (1.35–2.83)	2.74 *** (2.17–3.45)	2.44 *** (1.93–3.09)
	13	low	1.00 (ref)	1.00 (ref)	1.00 (ref)
		medium	1.24 (0.89–1.73)	1.26 * (1.03–1.53)	1.57 *** (1.29–1.9)
		high	1.78 ** (1.24–2.56)	1.83 *** (1.46–2.29)	2.65 *** (2.11–3.32)
	15	low	1.00 (ref)	1.00 (ref)	1.00 (ref)
		medium	1.86 *** (1.30–2.67)	1.46 *** (1.18–1.8)	1.68 *** (1.36–2.07)
		high	2.30 *** (1.55–3.41)	2.55 *** (2.01–3.24)	3.01 *** (2.35–3.87)

* *p* < 0.05, ** *p* < 0.01, *** *p* < 0.001. OR—odds ratio, CI—confidence interval, FAS—Family Affluence Scale. OR with *p* < 0.01 shown in bold numbers.

## Data Availability

The data that support the findings of this study are available from the corresponding author upon reasonable request.
